# Prediction of Back Disability Using Clinical, Functional, and Biomechanical Variables in Adults with Chronic Nonspecific Low Back Pain

**DOI:** 10.3390/jcm13133980

**Published:** 2024-07-08

**Authors:** Omar M. Elabd, Paul A. Oakley, Aliaa M. Elabd

**Affiliations:** 1 Department of Orthopedics and Its Surgeries, Faculty of Physical Therapy, Delta University for Science and Technology, Gamasa 35712, Egypt; 3omarel3abd@gmail.com; 2 Department of Physical Therapy, Aqaba University of Technology, Aqaba 771111, Jordan; 3 Private Practice, Newmarket, ON L3Y 8Y8, Canada; docoakley.icc@gmail.com; 4 Kinesiology and Health Science, York University, Toronto, ON M3J 1P3, Canada; 5 Basic Science Department, Faculty of Physical Therapy, Benha University, Benha 13511, Egypt

**Keywords:** back muscles, low back pain, postural balance, physical functional performance, chronic pain

## Abstract

**Background:** Researchers are focusing on understanding the etiology and predisposing factors of chronic nonspecific low back pain (CNSLBP), a costly prevalent and disabling disorder. Related clinical, functional, and biomechanical variables are often studied, but in isolation. We aimed to identify key factors for managing CNSLBP by examining the relationship between back disability and related clinical, functional, and biomechanical variables and developed prediction models to estimate disability using various variables. **Methods:** We performed a cross-sectional correlational study on 100 recruited patients with CNSLBP. Clinical variables of pain intensity (visual analog score), back extensor endurance (Sorenson test), functional variables of the back performance scale, 6 min walk test, and the biomechanical variable C7-S1 sagittal vertical axis were analyzed to predict disability (Oswestry disability index). **Results:** All variables independently, as well as in multi-correlation, were significantly correlated to disability (*p* < 0.05). The bivariate regression models were significant between back disability and pain intensity (Y = 11.24 + 2.189x), Sorensen results (Y = 105.48 − 0.911x), the back performance scale (Y = 6.65 + 2.486x), 6 min walk test (Y = 49.20 − 0.060x), and sagittal vertical axis (Y = 0.72 + 4.23x). The multi-regression model showed significant contributions from pain (*p* = 0.001) and Sorensen results (*p* = 0.028) in predicting back disability, whereas no significant effect was found for other variables. **Conclusions:** A multidisciplinary approach is essential not only for the management of but also for the assessment of chronic nonspecific low back pain, including its clinical, functional, and biomechanical characteristics. However, special emphasis should be placed on clinical characteristics, including the intensity of pain and back extensor endurance.

## 1. Introduction

Globally, low back pain (LBP) is the leading cause of years lived with disability and currently affects half a billion people worldwide [1]. Chronic LBP, a recurrent discomfort that lasts longer than three months [2], represents a growing global burden affecting 5–10% of patients [3]. There are research needs to reduce its impact on social and health systems [4]. Disability is a significant issue in LBP [5] impacting physical performance and work productivity. LBP causes disability, absenteeism, and economic burdens globally [6].

Research investigating the etiology and predisposing factors for nonspecific (i.e., idiopathic [7]) low back pain (NSLBP) is needed; however, the challenge lies in ignoring the role of dysfunction in LBP [8], as dysfunction often interacts with pathoanatomy to cause clinical symptoms [9]. Identifying the factors contributing to chronic (C)NSLBP-related disability is crucial for effective interventions [10]. Recent evidence suggests a multidisciplinary approach for optimal treatment [11], and Sirbu et al. [12] suggest that the optimal management of CNSLBP should involve mental, social, and physical evaluations.

Although the correlation between pain intensity and back disability has been consistently observed [13,14,15], functional measurements are often overlooked in LBP studies [16]. However, the need for functional tests in CNSLBP patients is obvious, particularly in occupational health services. Isometric trunk extensor endurance, for example, is a critical outcome measure [17,18] as lumbar extensor muscles play a crucial role in motor and postural control [19,20]. The back performance scale (BPS) is another promising functional test for evaluating return-to-work, focusing on mobility-related activities [21,22]. Additionally, the 6 minute walk test (6MWT) is a valuable tool highlighting the importance of walking endurance in overall health [23,24].

Biomechanical dysfunctions related to CNSLBP are influenced by the sagittal spinal geometry, with reported changes in sagittal alignment between LBP patients and those without LBP [25]. Changes in sagittal spinal alignment, such as the sagittal vertical axis (SVA: a horizontal distance between a plumb line between C7 and S1), may increase loads on spinal soft tissues and impact core stability, altering kinematics and load sharing on spinal components [26,27,28]. The current standard measurement for global sagittal alignment is the C7-S1 SVA [29]; however, it is not frequently used as an outcome measure for CNSLBP [30].

Key prognostic variables guide treatment and intervention strategies to improve outcomes in individuals with back disabilities. Silva et al.’s [31] systematic review highlighted the absence of a suitable prediction model for individuals with recent-onset LBP. Mukasa and Sung [32] developed and validated a prediction model for LBP risk but recommended incorporating other predictors in different settings. Petrozzi et al. [33] highlighted the need for alternative management strategies to prevent prolonged disability in patients with LBP by identifying the clinically relevant predictors.

All previously mentioned clinical, functional, and biomechanical variables may be crucial for managing CNSLBP; however, there is limited evidence examining their association with back disability. To fill this gap, this study examined the association between back disability measured by the Oswestry disability index (ODI), back pain intensity measured by the visual analog scale (VAS), back extensor endurance assessed by the Sorensen test, functional performance assessed by both the BPS and 6MWT, and a biomechanical variable quantifying sagittal balance by the C7-S1 SVA. Herein, we developed prediction models for estimating back disability in CNSLBP. We hypothesized all variables will be statistically significant predictors of disability.

## 2. Materials and Methods

This cross-sectional correlational study was carried out from November 2023 to January 2024 at the outpatient clinic of Benha University’s Faculty of Physical Therapy. This study aimed to analyze the correlations among the examined variables and create prediction models to estimate back disability based on these variables. This study adhered to the 1964 Declaration of Helsinki and the related subsequent corrections. It was registered at clinicaltrial.gov (NCT06186674) following approval from the Faculty of Physical Therapy Research Ethics Committee (PT. BU. EC.1). Participation in the study was voluntary. Written informed consent was obtained from each participant prior to inclusion. Patients’ data were anonymized and protected. Patient identifier data were collected by an independent researcher not involved in the research. Research data were protected by creating a dataset that was updated regularly. Access to the dataset of all patients was available only to the researcher; also, each patient was given his/her own data in a file separate from the research dataset.

### 2.1. Participants

Participants were recruited through a university email campaign targeting adult office workers and university students with CNSLBP. The diagnosis was made by an orthopedist after a standardized physical examination [34] and screening for eligibility by a researcher with 10 years of experience. One hundred patients met the eligibility requirements, signed the consent form, and took part in the study.

Inclusion criteria included those aged 20 to 40 years [35], either sex, mild-to-moderate [36] disability up to 40% on the ODI, and chronic symptoms (>3 months) [2]. Exclusion criteria included those with congenital spinal deformity, scoliosis, visual/auditory issues, neurological disorders, inflammatory diseases, spinal surgery, or pathologies including neoplasms, dislocations, fractures, discogenic radiculopathy, and/or degenerative changes.

### 2.2. Procedure

Demographic variables (age, weight, height, BMI, and sex) were collected on the day of inclusion. The ODI was used as a dependent (predicted) variable with five independent (predictor) variables: clinical (VAS; Sorensen test), functional performance (BPS; 6MWT), and biomechanical (C7-S1 SVA).

The ODI was used to quantify functional disability and has acceptable validity and reliability [36]. The original English version of the ODI was used. The patient was asked to tick one box for each of ten categories, each scored on a 0–5 rating scale, corresponding to the most accurate statement; the score was calculated using the formula (counted marks/50 × 100%). Disability can be interpreted as 0–20% mild disability, 21–40% moderate disability, 41–60% severe disability, 61–80% crippled handicap, 81–100% complete disability [37]. The standard error of measurement for the ODI is up to 10% [38].

The VAS was used to assess the pain intensity and is reliable, effective, and widely used [39]. With “no pain” on the left and “worst imaginable pain” on the right of a 10 cm VAS line, patients indicated their level of discomfort with scores calculated from the left end to the designated point. The SEM of the VAS ranges from about 9 to 14 mm [38,40]. Notably, both the ODI and VAS questionnaires were given in a standardized presentation. They were well described and explained prior to being given. Also, the questionnaires were measured in a continuous way instead of at a single time point; they were measured 4 times a day (once after waking up, once before going to the bed, and twice during the day), and the average was calculated.

The Biering-Sorensen test was used to assess trunk extensor muscle endurance and has demonstrated reliability and validity [41]. The patient was positioned prone on a treatment bench, securely strapped at the greater trochanter, knee creases, ankles, and below the anterior superior iliac spines. The test involved patients resting their upper body on a chair, raising it off, crossing their arms, and maintaining a neutral trunk alignment. The examiner then calculated the time spent in this position. Ending the test was possible because of fatigue, intense pain, or an inability to maintain a neutral trunk position [17,41,42].

Physical performance was assessed using the BPS and 6MWT. There are five examinations in the BPS that measure trunk mobility related to daily tasks: sock test, the patient attempted to put on a sock in an optional manner while seated; pick-up test, the patient attempted to pick up a piece of paper from the ground in a predictable manner; roll-up test, the patient attempted to roll from a supine position to an extended sitting position slowly and with relaxed arms; the fingertip-to-floor test involves bending forward with straight knees to touch the floor with fingertips, using centimeters to measure the distance between the middle finger and the ground; and lift test, the patient was asked to lift a 5 kg sandbag-containing box repeatedly for one minute, moving it from the floor to a table and back again [21,43,44]. The BPS sum score ranges from to 0 to 15, where higher scores denote limited performance. The BPS has strong internal consistency, discriminating power, and adaptability, making it valid and trustworthy for evaluating back pain performance [21,43,44].

The 6MWT is a valid and reliable method of measuring bodily functioning [45]. It calculates the maximum distance a person can walk on a level, hard surface in 6 min. Cones were positioned at the start and finish of a 30 m border to signify turns, and interval marks were utilized every three meters in an indoor, 30 m walkway. The objective was to walk as far as possible at a self-selected pace for 6 min. Before the examination, patients were instructed to wear comfortable shoes and clothes. Standardized words of encouragement were delivered [24,45].

The C7-S1 SVA served as a sagittal spine alignment biomechanical predictor and was calculated as the distance from the plumb line dropped from the centroid of a C7 vertebral body to the posterior–superior corner of S1 [29]. A lateral-view radiograph of the entire spine was obtained using a 72-inch film focal distance using a Toshiba Rotanode (model: DRX3724HD/2009, Benha, Egypt) with the patient standing in a neutral position with the hands loosely clasped with arms relaxed in front [46]. The assessor used AutoCAD software (version 2017; Mill Valley, CA, USA) to measure the C7-S1 SVA as the validity and reliability were established [47,48,49]. Ten radiographs were examined twice by the same assessor (separated by 1 week) to evaluate the intra-rater reliability of the C7-S1 SVA measurements and showed excellent intra-rater reliability (ICC = 0.91). The SEM of the SVA was found to be approximately 5–10 mm [47].

### 2.3. Statistical Analysis

Statistical analyses were performed using SPSS for Windows (v.25 SPSS, Inc., Chicago, IL, USA). The Shapiro–Wilk test was used to test the normality of the data distribution of each of the variables. Bivariate Pearson correlation analysis was used to compute the relationship and direction between back disability and pain intensity and Sorensen, BPS, 6MWT, and C7-S1 SVA variables. A multi-correlation analysis was used to compute the relationship between the five examined independent variables and back disability. Bivariate regression analysis was used to compute the best-fit model for disability, using the examined variables. A multi-regression analysis was used to compute the best-fit model between the examined variables and back disability. A sample size estimation was determined for performing the multiple regression analysis; assuming an 80% power, a significance level of 95% (*p* < 0.05), and a moderate effect size (Cohen’s *f*^2^ = 0.15), a sample of *n* = 91 was necessary (https://www.danielsoper.com/statcalc/calculator.aspx?id=1, accessed on 12 April 2024). All other statistical tests also utilized a significance level of *p* < 0.05.

## 3. Results

Of the 128 participants in the baseline survey, 28 were excluded (15 did not satisfy inclusion, 10 were receiving treatment for LBP, and 3 declined to participate). A total of 100 patients participated in this study (56 female/44 male). All data were normally distributed according to the Shapiro–Wilk test. The mean ± SD (minimum–maximum) values in the study population group were for age 33.0 ± 6.0 years (22.0–40.0 years), weight 79.4 ± 10.4 kg (57.0–95.0 kg), height 171.3 ± 7.3 cm (160.0–185.0 cm), and BMI 27.0 ± 2.0 kg/m^2^ (22.3–29.4 kg/m^2^) (Table 1). There were no missing data.

**Figure 1:**
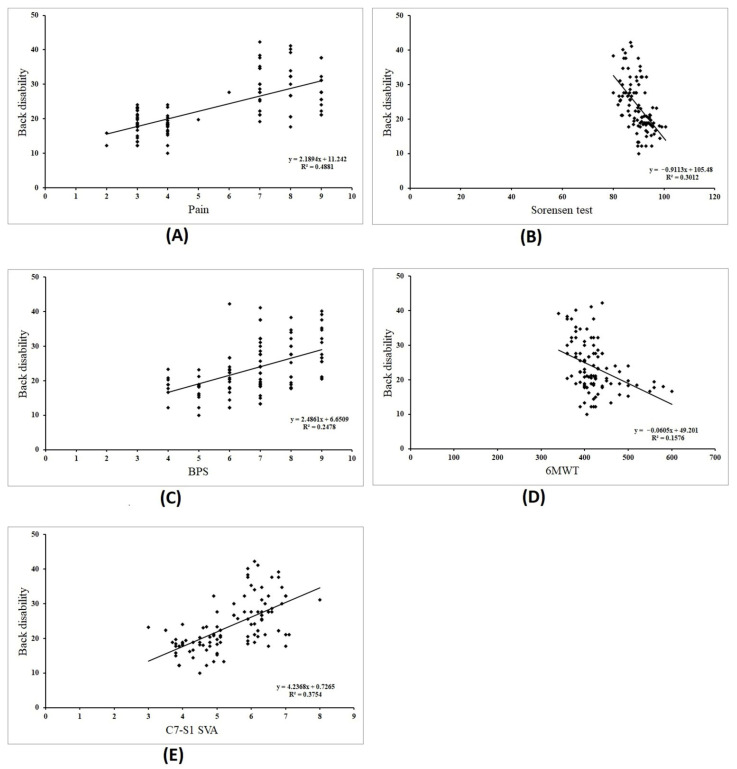
Scatter plot correlations between back disability and the examined variables of pain intensity (**A**), the Sorensen test (**B**), back performance scale (BPS, (**C**)), 6 min walk test (6MWT, (**D**)) and C7-S1 sagittal vertical axis (C7-S1 SVA, (**E**)).

A strong positive correlation was found between back disability and pain (r = 0.699; *p* = 0.0001), BPS (r = 0.501; *p* = 0.0001), and C7-S1 SVA (r = 0.613; *p* = 0.0001), a strong negative correlation with Sorensen (r = −0.549; *p* = 0.0001), and a moderate negative correlation with the 6MWT (r = −0.397; *p* = 0.0001) (Figure 1). Furthermore, this study found a significant positive multi-correlation (r = 0.723; *p* = 0.0001) between all the measured variables and back disability (Table 2).

Bivariate linear regression analyses (Table 3, Figure 1) showed a significant predictor of back disability and pain (Y = 11.24 + 2.189x), indicating that for every additional unit in pain (centimeter on the VAS), back disability increased by an average of 2.189%. Furthermore, the regression model revealed a significant predictor (*p* = 0.0001) between back disability and Sorensen scores (Y = 105.48 − 0.911x), indicating that for every additional second in the Sorensen score, we can expect back disability to decrease by an average of −0.911%. The fit regression models between back disability and the BPS (Y = 6.65 + 2.486x), 6 MWT results (Y = 49.20 − 0.060x), and C7-S1 SVA (Y = 0.72 + 4.23x) are significant predictors (*p* = 0.0001). The models predict that for every additional unit in the BPS (point), 6MWT (meter), and C7-S1 SVA (centimeter), back disability increased by an average of 2.486%, decreased by an average of −0.060%, and increased by an average of 4.23%, respectively.

The multi-regression equation (Table 4) between the disability and examined variables revealed that the fit model (Y = 46.84 + 1.540x_1_ + 0.339x_2_ + 0.050x_3_ + 0.010x_4_ + 0.541x_5_) was significant (*p* = 0.0001). The results of the multi-regression fit model to predict back disability indicated significant contributions from pain (*p* = 0.001) and Sorensen (*p* = 0.028), while there were no significant effects due to the BPS (*p* = 0.921), 6MWT (*p* = 0.439), or C7-S1 SVA (*p* = 0.542).

## 4. Discussion

This study examined back disability and associated clinical, functional, and biomechanical factors in patients with CNSLBP and predicted disability based on the examined variables. The results support our hypothesis and highlight the correlation between back disability with clinical variables of back pain intensity and back extensor endurance, functional performance indicated by the BPS and 6MWT, and the biomechanical variable of sagittal spinal alignment indicated by the C7-S1 SVA. Furthermore, it revealed that each variable was a significant predictor of back disability.

However, only the clinical factors (pain intensity and back extensor endurance) in the multi-regression model for back disability prediction contributed significantly to the multi-regression model for the prediction of disability in patients with CNSLBP. This is not surprising, as most items on the Oswestry disability index focus on clinical suffering during functional activities, particularly those requiring the constant contraction of the trunk extensor muscles. In agreement with the current results, previous studies have suggested that pain intensity and trunk extensor endurance are significant predictors of disability in populations with CNSLBP [12,15,20,50,51]. The capacity of the trunk muscles to shield spinal structures from damage in patients with CNSLBP can be hampered by time-dependent functional alterations in soft tissues causing physiological, anatomical, and behavioral changes [52,53,54].

Despite advancements in medical knowledge and treatment, CNSLBP remains a significant global health issue, and researchers continue to prioritize its clinical, societal, and economic burdens. All stakeholders in CNSLBP management aim to allocate limited healthcare resources for discovering clinically beneficial assessment and treatment methods [3,4,6]. Recent studies have focused on predicting back disability and examining related variables, but functional and biomechanical variables are often overlooked [12,31,32,33]. To the best of our knowledge, this study is the first to explore the correlation between back disability and various clinical, functional, and biomechanical factors and predict changes in back disability based on these variables. The variables examined were affordable, simple to use, and had important predictive roles, making them suitable for use as outcome measures in clinical practice [55].

Regarding the biomechanical aspect, the strong correlation revealed between back disability and the biomechanical variable C7-S1 SVA could be attributed to the fact that spinal alignment can influence the load location and magnitude of spinal components as well as alter kinematics and load sharing [28,56]. This suggests a focus on subject-specific spinal sagittal organization in CNSLBP patients’ mechanical analysis. Further, the C7-S1 SVA can be a very successful discriminator between CLBP subjects and healthy controls with the patients showing forward sagittal balance [25]. Recently, it has been shown that although pelvic morphology is similar between normal and CLBP patients, CLBP patients had an abnormal ‘fit’ of their lumbar lordosis to their pelvic morphology and sacral tilt as demonstrated as hypolordosis; thus, not surprisingly, biomechanical parameters are an emerging factor of importance in the discrimination between non-LBP and LBP groups [57]. Therefore, it could be hypothesized that global spinal sagittal alignment, postural correction, and exercises can reduce postural aberrations. This agrees with previous studies that suggest the integration of biomechanical corrections for LBP [58,59].

On the other hand, this study’s findings emphasize the significance of functional physical performance in contributing to the disability burden in CNSLBP. Physiological alterations in motor patterns and decreased overall performance could account for the loss of physical capacities caused by back pain instead of only specific spinal deficits [21]. Further, it has been shown that in people with CNSLBP, balance, trunk muscular endurance, and functional performance levels are related. Decreased stabilization may affect the body’s ability to maintain adjustments and balance reactions [60,61]. Therefore, this study’s results emphasize the need for not only a multidisciplinary treatment approach but also a multimodal assessment approach that considers clinical, functional, and biomechanical factors. This agrees with a systematic review by Lin et al. [62] which highlights the link between physical activity and disability in LBP patients. The recommendation is to implement interventions aimed at enhancing physical activity levels in patients with CNSLBP [63].

The limitations of this study include the lack of the generalizability of the findings to older age groups since this cohort was limited to a young adult cohort ranging in age from 22 to 40 years. Furthermore, a control group of asymptomatic participants was not included. Another limitation is that neither the frontal nor the transverse plane parameters were considered when examining postural alignment. Also, the physical activity level was not controlled for. Additionally, only the standing posture was used for biomechanical measurements; sitting or other functional positions were not considered. Finally, the psychosocial aspects of CNSLBP are important, and further research should include such measures, as well as those included herein. The strengths of this study include using diverse assessment domains including clinical, functional, and biomechanical measures which are rarely all considered in the prediction of disability in CNSLBP patients. Also, the independent predictor variables used in this study represent fairly simple and cost-effective means to assess the clinical, functional, and biomechanical aspects of a patient’s health status and are easy to implement in clinical practice. Future research should be conducted in an older age group and include patients with greater disability.

## 5. Conclusions

This study revealed that dysfunctions such as faulty posture, limited back extensor endurance, and restricted functional performance, not just lumbosacral pathoanatomy, significantly correlated with and predicted back disability in patients with CNSLBP. This study revealed that clinical variables, specifically pain intensity and back extensor endurance, were significantly correlated with back disability in patients with CNSLBP, according to a multi-regression model. Thus, a multidisciplinary strategy incorporating clinical, functional, and biomechanical aspects is crucial for assessing patients with CNSLBP. However, clinical features such as back extensor endurance and pain intensity should be given particular attention.

## Figures and Tables

**Table 1:** The socio-demographic characteristics and Oswestry index of the study population group (*n* = 100).

Variables	Mean (SD)	Minimum	Maximum
Age (year)	33.0 (6.0)	22.0	40.0
Weight (kg)	79.4 (10.4)	57.0	95.0
Height (cm)	171.3 (7.3)	160.0	185.0
BMI (kg/m^2^)	27.0 (2.0)	22.3	29.4
ODI (%)	23.7 (7.4)	10	42.2

Note: Data are expressed as mean (standard deviation, SD); ODI: Oswestry disability index.

**Table 2:** Bivariate and multi-correlation between back disability with clinical, functional, and biomechanical variables.

Variables	Mean (SD) (n = 100)	r	*p*-Value ^1^	r (*p*-Value ^2^)
Pain (cm)	5.67 ± 2.36	0.699	0.0001 *	0.723 (0.0001 *)
Sorensen (seconds)	89.79 ± 4.45	−0.549	0.0001 *	
BPS (points)	6.84 ± 1.48	0.501	0.0001 *	
6MWT (m)	422.24 ± 48.56	−0.397	0.0001 *	
C7-S1 SVA (cm)	5.41 ± 1.07	0.613	0.0001 *	

Data are expressed as mean ± standard deviation; r: Pearson’s correlation coefficient; * significant: *p* > 0.05; *p*-value ^1^: probability value for bivariate correlation coefficient; *p*-value ^2^: probability value for multi-correlation coefficient.

**Table 3:** Bivariate regression analysis between back disability (dependent variables, Y) with clinical, functional, and biomechanical variables (independent variables, x).

Dependent Variables (Y)	Independent Variable (x)	Bivariate Regression Model	95% CI	*p*-Value
Back disability	Pain	Y = 11.24 + 2.189x	1.74–2.63	0.0001 *
	Sorensen	Y = 105.48 − 0.911x	−1.19–−0.63	0.0001 *
	BPS	Y = 6.65 + 2.486x	1.61–3.35	0.0001 *
	6MWT	Y = 49.20 − 0.060x	−0.08–−0.03	0.0001 *
	C7-S1 SVA	Y = 0.72 + 4.23x	3.14–5.33	0.0001 *

*p*-value: probability value for bivariate regression; *p*-value < 0.05 significant; * significant (*p* < 0.05) 95% confidence interval.

**Table 4:** Multivariate regression analysis among back disability (dependent variables, Y) with clinical, functional, and biomechanical variables (independent variables, x).

Dependent Variables (Y)	Independent Variable (x)	Multivariate Regression Model	95% CI	*p*-Value ^1^	*p*-Value ^2^
Back disability	Constant	46.84	14.52–79.16	0.005 *	0.0001 *
	Pain (x_1_)	1.540	0.61–2.46	0.001 *	
	Sorensen (x_2_)	−0.339	−0.64–−0.04	0.028 *	
	BPS (x_3_)	−0.050	−1.04–0.94	0.921	
	6MWT (x_4_)	−0.010	−0.03–0.02	0.439	
	C7-S1 SVA (x_5_)	0.541	−1.21–2.29	0.542	

*p*-value ^1^: probability value for each independent variable; *p*-value ^2^: probability value for the multivariate regression model. *p*-value > 0.05: non-significant; *p*-value < 0.05: significant; * significant (*p* < 0.05) 95% confidence interval.

## Data Availability

Additional pertinent data are available upon reasonable request.
